# Auditory Mismatch Responses to Emotional Stimuli in 3-Year-Olds in Relation to Prenatal Maternal Depression Symptoms

**DOI:** 10.3389/fnins.2022.868270

**Published:** 2022-05-17

**Authors:** Silja Luotonen, Henry Railo, Henriette Acosta, Minna Huotilainen, Maria Lavonius, Linnea Karlsson, Hasse Karlsson, Jetro J. Tuulari

**Affiliations:** ^1^Turku Brain and Mind Center, Institute of Clinical Medicine, University of Turku, Turku, Finland; ^2^Department of Clinical Neurophysiology, University of Turku, Turku, Finland; ^3^Department of Psychiatry and Psychotherapy, Philipps University of Marburg, Marburg, Germany; ^4^CICERO Learning, Faculty of Educational Sciences, University of Helsinki, Helsinki, Finland; ^5^Cognitive Brain Research Unit, Faculty of Medicine, University of Helsinki, Helsinki, Finland; ^6^Centre for Population Health Research, Turku University Hospital, University of Turku, Turku, Finland; ^7^Department of Psychiatry, Department of Clinical Medicine, Turku University Hospital, University of Turku, Turku, Finland; ^8^Turku Collegium for Science, Medicine and Technology, University of Turku, Turku, Finland; ^9^Department of Psychiatry, University of Oxford, Oxford, United Kingdom

**Keywords:** EEG, mismatch response, brain, child, maternal depression, auditory

## Abstract

Maternal depression symptoms are common in pregnant women and can have negative effects on offspring’s emotional development. This study investigated the association between prenatal maternal depression symptoms (assessed with the Edinburgh Postnatal Depression Scale at 24 weeks of gestation) and auditory perception of emotional stimuli in 3-year-olds (*n* = 58) from the FinnBrain Birth Cohort Study. Using electroencephalography (EEG), we examined mismatch responses for happy, sad, and angry sounds presented among neutral stimuli. A positive association between maternal depression symptoms and the emotional mismatch responses in an early time window (80–120 ms) was found, indicating that brain responses of children of mothers with depressive symptoms were weaker to happy sounds, though the results did not survive Bonferroni correction. There were no clear associations in the sad and angry emotional categories. Our results tentatively support that the 3-year-old children of mothers with depression symptoms may be less sensitive to automatically detect happy sounds compared to children whose mothers do not display symptoms of depression.

## Introduction

Prenatal stress can be defined as maternal prenatal psychological symptoms of depression or anxiety, or perceived stress related to daily hassles or major life events (Karlsson et al., 2018). It is an important early-life stressor (Karlsson et al., 2018) and common in pregnant women (Dunkel Schetter and Tanner, 2012). Prenatal stress increases the offspring risk for different mental and behavioral disorders (Tuovinen et al., 2021) by modulating the child brain development (Räikkönen et al., 2011), leading to a need to investigate further the possible associations between the prenatal stress and the neural processing in offspring.

Prenatal stress may affect the developmental processes of the fetus, and these effects are influential over the life span (Räikkönen et al., 2011). Developmental Origins of Health and Disease hypothesis (DOHaD, Barker, 1998) suggests that exposure to adverse environmental experiences, such as prenatal stress, during critical periods of early development may permanently modulate the structure and function of cells, organs, and physiological systems (Räikkönen et al., 2011). The dysregulation of hypothalamic pituitary adrenal axis has been thought to be a possible mediator of prenatal stress on fetal brain (Huizink et al., 2004). The modulated programming of the fetus may change the developmental pathways of neurocognitive processes by altering the architecture of the neural cells (van den Bergh, 2011). *In vivo* brain imaging studies have reported consistent associations between prenatal stress and changes in offspring limbic and frontotemporal brain networks, as well as in connections linking them (for review, refer to Lautarescu et al., 2020). Maternal depression decreases functional connectivity between visualization and auditory regions (Farah et al., 2020). Furthermore, recent results from our group suggest that neural phenotypes are in turn associated with infant self-regulation (Nolvi et al., 2021), temperament (Nolvi et al., 2020), and visual emotional processing assessed via eye-tracking (Tuulari et al., 2020). In this study, we were interested in whether the exposure to prenatal stress, more specifically to maternal depression symptoms, could be associated with offspring processing of emotionally valanced sounds.

Newborns already show strong and rapid neural responses to emotional sounds (Kostilainen et al., 2018). The event-related potentials (ERPs) are excellent tools for studying those rapid neural responses, thanks to their ability to capture the dynamics of neuronal responses in the order of milliseconds (Leventon and Bauer, 2016). The ERPs are extracted from the electroencephalogram (EEG), typically by averaging stimulus-locked EEG responses (Duncan et al., 2009). One of the most commonly studied components of ERPs is the mismatch negativity (MMN), which is a change-specific component of the ERP reflecting the cortical discrimination of changes in a sequence of sounds (Näätänen et al., 2004). The MMN reflects automatic sound processing, and thus, it can be recorded regardless of where the subject focuses his or her attention to, and that makes it a valuable tool in cognitive neuroscience especially in young children (Putkinen et al., 2012). The main neural generators of MMN are located in the auditory cortices of the temporal lobes, and the peak of activation occurs typically at 100–250 ms after the deviance onset (Pakarinen et al., 2014). In addition to this classical, “early” MMN response, ERPs sometimes show also later MMN-like amplitude differences (Putkinen et al., 2012). In this study, we use the term “mismatch response” to describe all MMN-like responses, regardless of their time window. These mismatch responses (difference in the ERP response to deviant sounds compared to standard sounds) appear already in infancy and turn into relatively adult-like responses during school age (Putkinen et al., 2012).

Despite the recent good quality studies addressing the relation of prenatal stress and child emotional processing in infancy (Otte et al., 2015; Soe et al., 2016; Gustafsson et al., 2018; Goodman et al., 2021), the association of maternal prenatal stress with toddler’s neural processing of emotional information is unknown. Previous research, to our knowledge, has mainly focused on maternal prenatal anxiety as the indicator of prenatal stress (Otte et al., 2015; van den Heuvel et al., 2018). These studies suggest that the association between maternal anxiety and emotional perception of, e.g., fearful sounds and pictures in children. Otte et al. (2015) showed in their study that 9-month-old infants of anxious mothers showed stronger reactions to fearful sounds, independently from the emotion of the simultaneously presented visual stimuli. Furthermore, van den Heuvel et al. (2018) found that 4-year-olds, prenatally exposed to higher maternal anxiety, exhibited more attention to neutral pictures but not to unpleasant pictures. Correspondingly, maternal mindfulness during pregnancy seems to be associated with 9-month-old infants showing fewer attentional resources to neutral auditory stimuli (van den Heuvel et al., 2015).

Several studies have assessed the relation between maternal depression symptoms and the EEG of the children. However, prior studies have mainly focused on investigating functional measures such as frontal asymmetry derived from EEG in infants exposed to prenatal (Gustafsson et al., 2018), postpartum (Dawson et al., 1992, 1999; Lusby et al., 2014), or both prenatal and postpartum (Soe et al., 2016; Goodman et al., 2021) maternal depression. Even the subclinical levels of prenatal maternal depression may have potential effects on infant brain development and EEG (Gustafsson et al., 2018), and one potential trait marker of child’s vulnerability to maternal depression could be relative frontal EEG asymmetry (Goodman et al., 2021). This pattern of brain activity has been shown to generalize to various situations, for example, to positive interactions with non-depressed adults (Dawson et al., 1999). Prenatal maternal depression may better predict offspring’s EEG activity compared to postnatal or concurrent depression (Goodman et al., 2021). Furthermore, Porto et al. (2020), using fNIRS, showed that greater maternal negative affect (assessed postpartum) was associated with greater oxyhemoglobin activation in emotion-related brain region in infants, even if they did not find support for greater right versus left frontal cortex activation in association with maternal negative affect. Lavonius et al. (2020), using the same birth cohort data as we, found the association between higher maternal sleep loss during pregnancy and decreased ERP amplitude for happy sounds in infants.

There is still a clear paucity of ERP studies in toddlers and small children, and we are not aware of any studies that have investigated the links between prenatal maternal depression symptoms and emotional sound perception in toddlers.

In this study, we studied the association between mid-gestation prenatal maternal depression symptoms and emotional sound perception in 3-year-old children. We used mismatch response to measure the extent of difference of emotional auditory stimuli and to draw the focus of attention, and Edinburgh Postnatal Depression Scale (EPDS) to assess maternal depressive symptoms during pregnancy. We hypothesized that (1) maternal prenatal depressive symptoms are associated with the mismatch responses to emotional sounds in children and (2) this association varies with respect to the emotional category of auditory stimuli.

## Materials and Methods

### Participants

This cross-sectional study is a part of the FinnBrain Birth Cohort Study (^1^ Karlsson et al., 2018), a prospective population-based pregnancy cohort (*N* = 3,808 families), which is focused on investigating the effects of early-life stress on the brain development of the child using and combining different brain imaging techniques, molecular genetic studies, and neurophysiological assessments. Families of the cohort were recruited during the years 2011–2015 at the first trimester ultrasound (around gestational week 12) in the South-Western Hospital District and the Åland Islands, Finland. The FinnBrain Birth Cohort Study has been approved by the Ethics Committee of the Hospital District of Southwest Finland (ETMK: 57/180/2011, 15.3.2016§109), and the studies were conducted according to the Declaration of Helsinki.

Overall, 127 three-year-old children were recruited for an EEG recording from Southwest Finland during the years 2016–2018. Participants were born between 2013 and 2015. A written informed consent was collected from parents for their children to participate in this study.

**FIGURE 1:**
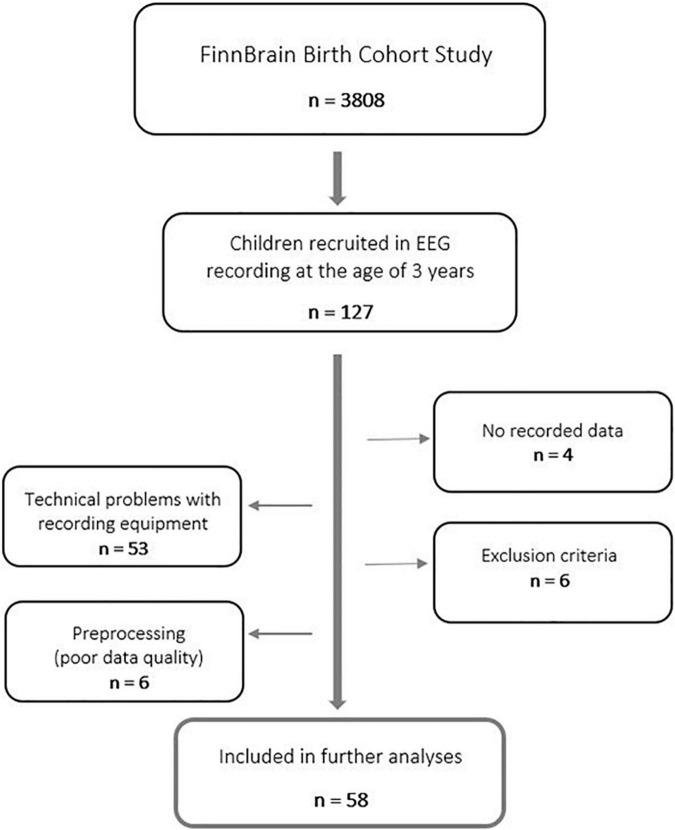
Study flowchart.

Data of four children were excluded because of difficulties in cooperation during the EEG recording (practically no recorded data at all). Furthermore, data of 53 participants were omitted due to unexpected, and for the time, unnoticed technical problems with the recording equipment. Therefore, only data recorded after maintenance of amplifier-related parts were used in the further analyses to guarantee data quality. These technical problems were not affecting the results in a biased way, though they concerned all participants equally. Finally, data of two participants were excluded based on maternal use of central nervous system (CNS) affecting medication during the first trimester of pregnancy, three participants based on missing information of maternal use of CNS medication during the first trimester of pregnancy, one participant based on gestational age less than 36 weeks, and one participant based on low Apgar score at 5 min after birth (<7). Further, data of five participants were excluded later in the preprocessing of data (insufficient data quality). In the end, data of 58 *n*-dyads were used in the final statistical analyses (Figure 1). For a detailed participant information, refer to Supplementary Table 1.

### Measures

#### Maternal Depression Symptoms

Self-reported data of maternal depression symptoms during pregnancy were collected at the gestational week 24 as well as 3, 6, 12, and 24 months postpartum. Depression symptoms were assessed with the Edinburgh Postnatal Depression Scale (EPDS), consisting of 10 items with a four-point Likert scale ranging from 0 to 3. A higher total score (range between 0 and 30) predicts a stronger possibility of clinical depression (Cox et al., 1987). EPDS has been shown to be a valid tool to screen depression symptoms during both antenatal and postnatal periods (Kozinszky and Dudas, 2015). A sum score for EPDS at the gestational week 24 were computed (missing values were replaced with the mean of other values, max three missing values per participant) and used as predictor in the analyses while EPDS sum scores at 3, 6, 12, and 24 months postpartum are reported as demographics in Supplementary Table 1.

#### Maternal Anxiety

To assess maternal anxiety symptoms during pregnancy, the self-reported data of maternal anxiety symptoms were collected at the gestational week 24 as well as 3, 6, and 24 months postpartum. The anxiety subscale from the Symptom Check-List (SCL-90) was used. The subscale consists of 10 items with a five-point Likert scale ranging from 0 to 4 (*not at all* to *extremely*) assessing symptoms associated with high manifest anxiety, for example, restlessness, nervousness, cognitive signs of anxiety, and panic attacks (Derogatis et al., 1976). A sum score for SCL-90 at the gestational week 24 were computed (missing values were replaced with the mean of other values, max three missing values per participant) and used as control variable in the analyses while SCL-90 sum scores at 3, 6, and 24 months postpartum are reported as demographics in Supplementary Table 1.

#### Stimuli

We used the multifeature MMN paradigm, originally developed by Näätänen et al. (2004) and further developed by adding the emotional stimuli variants by Pakarinen et al. (2014) [for the more detailed presentation of the employed experiment, refer to Kostilainen et al. (2018) and Lavonius et al. (2020)]. The paradigm includes a standard stimulus, four different types of linguistically relevant deviant stimuli, and three types of emotionally loaded uttered stimuli. A bisyllabic pseudo-word (/ta-ta/) was used as the standard stimulus (duration 336 ms, 46% probability, 200 trials in one recording). The linguistically relevant deviants differed from the standard stimulus either based on their slight changes in vowel duration, vowel change, intensity, or frequency. The emotional variants of (/ta-ta/) (angry, sad, and happy) occurred infrequently in the stimulus stream (duration 388, 337, and 436 ms, respectively, 3% probability each, 12 trials in one recording for each emotion). The sounds were presented with a stimulus onset asynchrony of 650 ms. The paradigm was run twice for each participant, resulting in a total amount of trials of 400 (standard) and 24 (for each emotion variant) per child.

#### Electroencephalogram Recording

The EEG of the children was recorded using a 32-channel electrode system (actiCAP electrode cap by EASYCAP, Germany) and BrainVision Quickamp amplifier (Brain Products, Germany). The electrode locations followed the international 10–20-system, and the sampling frequency was 250 Hz. The reference and ground electrodes were located on the forehead.

Before the EEG recording, a short story introducing the agenda of the visit (including a couple of pictures for a child to watch) was sent to every family via email to prepare the family and especially the child for the visit. The appointment for the recording was made with each family respecting their schedules, including options on both weekdays and weekends, as well as both early morning times and afternoon times. In addition, a teddy bear wearing the electrode cap was used to introduce the preparation process for the child and make him or her feel comfortable before starting the recording. During the preparation process (duration approximately 15 min), the investigator was applying the gel into the electrodes; meanwhile, the child was watching the animated series chosen by himself or herself.

To improve compliance and reduce motion artifacts during the EEG recording, children were watching a silent video while stimuli were presented. As a video, we used Inscapes-movie (Vanderwal et al., 2015) featuring abstract shapes but still minimizing cognitive load during the experiment. The Inscapes video was originally designed to improve compliance in functional MRI. In some cases, only if necessary, some toys and drawing tools were also given to a child to keep him or her feeling comfortable during the recording. The child was seated either alone on the chair or mother’s lap while the stimuli were presented from a loudspeaker located 1.25 m from the child with a volume of 60 dB. A room divider located between the child and the investigator was used to reduce the possible distractions. The paradigm was run twice for each child, and the child was offered a possibility to get a short break between those recording sessions (duration of one session was approximately 5 min), if needed. The total duration of the recording visit was approximately 45 min.

#### Event-Related Potential Measurement and Data Processing

The EEG data were preprocessed using MATLAB (r2017b) with EEGLAB toolbox (v13.5.4b) (Delorme and Makeig, 2004).

We used the PREP (Early Stage Preprocessing, Bigdely-Shamlo et al., 2015) pipeline to preprocess the data. The PREP pipeline is a standardized preprocessing pipeline created to remove unwanted artifacts and contaminants of noisy data channels during the preprocessing. Following the pipeline, the line frequency (in Finland 50 Hz) with its harmonics was removed. The data were referenced using the robust average reference algorithm.

Data were epoched to −100 to 550 ms segments with −100 ms to 0 baseline correction. A high-pass filter of 1 Hz was used. Artifactual trials were removed using the pop_jointprob function with local and global thresholds set to 4. Also, trials with transients exceeding ± 150 μV at any recording electrodes were rejected. Participants with less than 10/24 (41.6%) trials remained after rejection were excluded from further analyses. Finally, each participant had on average 19.2 valid trials for the angry stimulus (SD 5.72), 18.5 trials for the sad stimulus (SD 7.01), and 18.2 trials for the happy stimulus (SD 5.86) remained.

**FIGURE 2:**
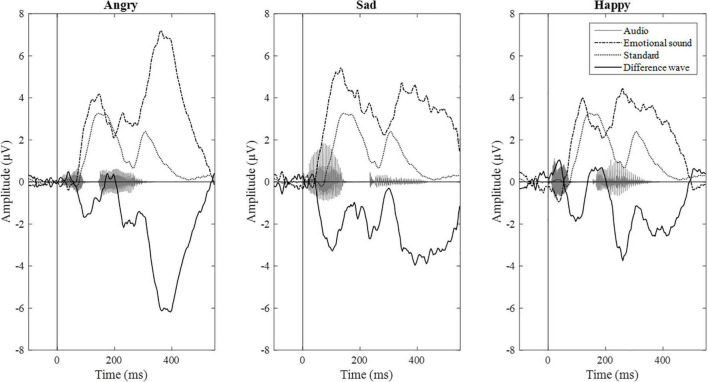
ERP waveforms from F-electrodes (F3, Fz, and F4).

**FIGURE 3:**
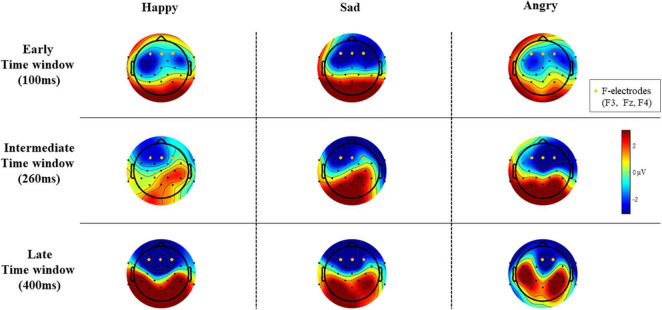
The difference wave amplitudes (mismatch responses) of all EEG electrodes in each time window (μV).

Epochs for each stimulus type were averaged separately, and the difference wave (standard – deviant; mismatch response) was calculated for each participant and each emotional stimulus. Difference waves with amplitudes exceeding ± 50 μV were rejected. By visual inspection, we chose three electrodes (F3, Fz, and F4) for further analyses based on their most negative difference between standard and emotional sounds throughout the recording. The decision was also based on previous data showing that MMNs can frequently be seen as negative displacement at the frontocentral and central electrodes (Näätänen et al., 2007). Then, the mean mismatch response amplitude values of the three frontal electrodes were averaged together (“F-electrodes”) and separately calculated for three different time windows, chosen by visual inspection. Time windows around three prominent mismatch responses were chosen, namely, 80–120 ms (early time window), 240–280 ms (intermediate time window), and 350–450 ms (late time window). ERP waveforms are presented in Figure 2 and the difference wave amplitudes (mismatch responses) in each time window in Figure 3.

#### Statistical Analyses

Statistical analyses were performed using MATLAB (r2017b, The MathWorks, Inc.) and IBM SPSS Statistics 25. First, in descriptive statistics we included sex of the child, native language, daycare status, maternal smoking/drinking/use of drugs during pregnancy, maternal educational level, and monthly income as categorical variables, as well as gestational weeks, Apgar points at 5 min, birth weight, age of child at the EEG recording (months), and number of siblings (at the age of 4 years). Also, age of the mother at delivery (years), maternal body mass index (kg/m^2^), depression symptoms (EPDS at gestational weeks 24 and 3, 6, 12, and 24 months postpartum) and maternal anxiety (SCL-90 at gestational weeks 24 and 3, 6, and 24 months postpartum) were computed. When reporting demographics, we followed recommendations reviewed by Pulli et al. (2019).

Second, a two-tailed one-sample *t*-test was used to assess the amplitude of the mismatch responses, determining whether the mean mismatch response amplitude values differed significantly from 0 μV in each of three different time windows (i.e., whether there was a statistically significant difference between ERP amplitudes to standard sound vs. to deviant sounds).

Finally, to assess if mismatch response amplitudes to emotional sounds (measured from the child) could be predicted using maternal prenatal depressive symptoms, the *fitlme* function of MATLAB was used to perform a linear mixed effects regression model analysis. Maternal depression symptoms (assessed with EPDS at gestational week 24), age of the child at measurement (months), sex of the child and the emotional category of the stimulus (happy, sad, and angry), as well as their interaction terms with maternal depression symptoms were entered in the model as fixed effects. The intercepts for subject were modeled as random effects. The statistical models were repeated for each time window (early/intermediate/late). The linear regression model had the following structure:

*Mismatch response*∼ *Depression* + *Angry* × *Depression* + *Sad* × *Depression* + *Sex* × *Depression* + *Age* × *Depression* + *(1/ID)*

*Mismatch response* is the mean mismatch response amplitude of the child for emotional stimuli in the selected time window. *Depression* means the maternal depression symptoms assessed with EPDS at gestational week 24, *Angry* and *Sad* mean the corresponding, dummy-coded emotion categories (angry/sad), *Sex* means the sex of the child, and *Age* means the age of the child at the measurement (months). *(1/ID)* is the random-effect structure, indicating that intercept was allowed to vary between participants. The emotional category “happy” was the baseline category (i.e., intercept is the mismatch response to happy stimuli). Random-effect structures that included inter-participant variation in all emotion categories did not improve the fit of the model [based on Akaike information criterion (AIC)], and therefore we report the results of the random-intercept model (the results remained the same despite the use of more complex random-effect structure). The age of child at the measurement was *z*-scored before being included in the model.

The structure of the linear regression model was chosen by trying different combinations of either only the main effect of *Sex/Age* or also their interaction terms with *Depression* in the model and then selecting the models with lowest AIC values. The model remained stable when only the fixed effect of *Age* or also its interaction term with *Depression* was included in the model (no significant difference between those two models tested with likelihood ratio test). The model described above was chosen. The visual inspection of residual plots did not reveal any significant deviations from normality. The outliers were detected by visual inspection of residual plots separately in each time window and were excluded from the regression model (*n* = 3 in early, *n* = 2 in intermediate, and *n* = 3 in late time window).

First, to control for the possible effect of maternal anxiety (assessed with SCL-90 at gestational week 24), maternal anxiety was included as a fixed effect in the model. However, there were no significant main effects of maternal anxiety or its interactions with depression symptoms in any of the three time windows, so maternal anxiety was left out of the final regression model. Second, to control for possible three-way interaction effects, the interaction between *Sex*, *Depression*, and *Angry/Sad*, as well as the interaction between *Age*, *Depression*, and *Angry/Sad*, was included in the model. However, no statistically significant interactions were revealed, so these interaction terms were left out of the final regression model. Finally, the models were also repeated using the sum score *Distress* [(SCL-90 score + EPDS score) at gestational week 24] instead of *Depression*. However, there was no statistically significant main effect of the *Distress* sum score for the child mismatch response amplitudes.

**FIGURE 4:**
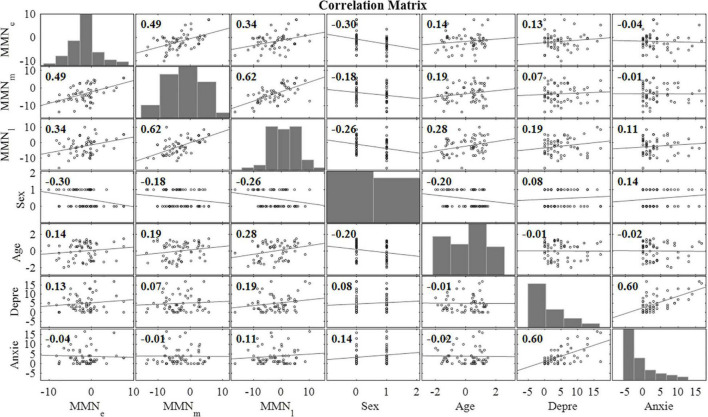
Correlation matrix for the main variables of interest (*n* = 58). MMN_e_ = mean mismatch response value (over all emotional categories) in early time window (80–120 ms), MMN_m_ = mean mismatch response value (over all emotional categories) in intermediate time window (240–280 ms), MMN_l_ = mean mismatch response value (over all emotional categories) in late time window (350–450 ms), *Sex* = the sex of the child, *Age* = the age of the child at the measurement (months), *Depre* = the maternal depression symptoms assessed with EPDS at gestational week 24, *Anxie* = the maternal anxiety symptoms assessed with SCL-90 at gestational week 24.

The correlation matrix of variables included in linear regression model analyses is presented in Figure 4.

## Results

### Mismatch Responses

The mean mismatch response amplitude values differed significantly from 0 μV for each emotional stimulus category in each time window (Table 1).

**TABLE 1:** Results of the two-tailed one-sample *t*-tests (*n* = 58).

Time latency	Emotion	mean (μv)	SD	95%CI	*t*-value (df)	*p*-value
**80–120 ms**						
	Happy	−1.68	3.59	−2.62…−0.73	−3.55 (57)	0.001
	Sad	−2.97	2.81	−3.71…−2.24	−8.07 (57)	≤0.001
	Angry	−1.45	3.24	−2.30…−0.60	−3.41 (57)	0.001
**240–280 ms**						
	Happy	−3.24	4.73	−4.49…−2.00	−5.22 (57)	≤0.001
	Sad	−2.00	4.82	−3.27…−0.73	−3.16 (57)	0.003
	Angry	−1.89	4.03	−2.95…−0.83	−3.57 (57)	0.001
**350–450 ms**						
	Happy	−2.28	5.48	−3.72…−0.84	−3.17 (57)	0.002
	Sad	−3.50	4.65	−4.72…−2.28	−5.73 (57)	≤0.001
	Angry	−5.25	4.65	−6.48…−4.03	−8.60 (57)	≤0.001

*Difference of the mean mismatch response amplitude values from 0 μV for the F-electrodes (F3, Fz, and F4).*

### Linear Mixed-Effects Regression Model

#### Early Time Window 80–120 ms

The results of the mixed-effects regression model on mismatch responses in the early time window are presented in Table 2. In the early time window, maternal depression symptoms were positively associated with the mismatch response amplitudes of the child [β = 0.213, 95% CI [0.0302, 0.396], (SE 0.0925), *p* = 0.0223, Bonferroni *p* = 0.0669]. That is, the increase of one point in the maternal EPDS sum score predicted a + 0.21 μV change in the mismatch response amplitude of the child. Note that because the mismatch response amplitude is a negative amplitude difference, the positive correlation indicates a decrease in mismatch response amplitude as maternal depression symptoms increase. As described below, interaction effects showed that this correlation applies especially to the happy emotion condition.

**TABLE 2:** Results of the linear mixed-effects regression model (DF = 162) in the early time window (80–120 ms).

Parameter	β	SE	tStat	*p*-value	95% CI
Happy (Intercept)	−2.461	0.630	−3.907	1.375	−3.705…−1.217
Sex	−0.408	0.765	−0.534	0.594	−1.919…1.103
Age	−0.051	0.389	−0.132	0.895	−0.820…0.717
Angry	1.734	0.704	2.463	0.015	0.344…3.125
Sad	0.248	0.703	0.353	0.725	−1.141…1.637
Depression	0.213	0.093	2.301	0.023	0.030…0.396
Sex × Depression	−0.164	0.111	−1.482	0.140	−0.383…0.055
Age × Depression	0.012	0.062	0.191	0.849	−0.110…0.134
Angry × Depression	−0.200	0.104	−1.923	0.056	−0.405…0.005
Sad × Depression	−0.246	0.104	−2.370	0.019	−0.451…−0.041

Furthermore, in the sad emotion category, the association between the mismatch responses and maternal depression symptoms was statistically significant [*Depression* × *Sad*: β = –0.246, 95% CI [–0.451, –0.0409], (SE 0.104), *p* = 0.0190, Bonferroni *p* = 0.0570]. This means that, in the sad emotion category, the increase of one point of the maternal EPDS sum score predicted a negligible –0.03 μV change in the MMN amplitude, taking into account the positive main effect of depression symptoms.

There was also a nearly statistically significant negative association between the mismatch responses and maternal depression symptoms in the angry emotion category [*Depression* × *Angry*: β = –0.200, 95% CI [–0.405, 0.00543], (SE 0.104), *p* = 0.0563]. Taking into account the positive main effect of depression symptoms, the increase of one point of the maternal EPDS sum score predicted the negligible 0.01 μV change in the mismatch response amplitude. In addition, we observed a statistically significant main effect of the angry emotion condition, showing weaker mismatch response amplitudes in the angry condition compared to the non-angry conditions [β = 1.73, 95% CI [0.344, 3.12], (SE 0.704), *p* = 0.0148, Bonferroni *p* = 0.0444].

**FIGURE 5:**
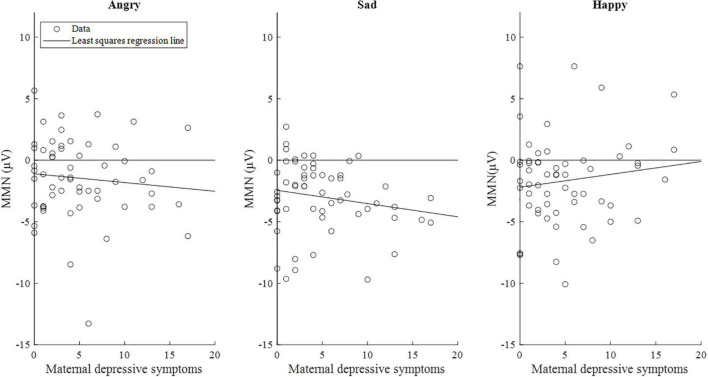
Scatterplots of maternal depressive symptoms and the mismatch response amplitudes (μV) of the children (*n* = 58) in the early time window (80–120 ms). Of note, the lines are least-squares regression lines fitted across the data points, not the results of the mixed-effects models.

The correlations between maternal depression symptoms and the mismatch response amplitudes of the child in the early time window are presented in Figure 5. Supplementary Figure 1 shows the results separately for girls and boys.

#### Intermediate Time Window 240–280 ms

The results of the mixed-effects regression model on mismatch responses in the intermediate time window are presented in Table 3. No statistically significant effects for the association of maternal depression symptoms and child mismatch response amplitudes were found in this time window. However, we observed a statistically significant main effect of the angry emotion condition, showing weaker mismatch response amplitudes in the angry condition compared to the non-angry conditions [β = 2.34, 95% CI [0.450, 4.23], (SE 0.957), *p* = 0.0156, Bonferroni *p* = 0.0468].

**TABLE 3:** Results of the linear mixed-effects regression model (DF = 162) in the intermediate time window (240–280 ms).

Parameter	β	SE	tStat	*p*-value	95% CI
Happy (Intercept)	−3.729	0.947	−3.940	1.208	−5.598…−1.860
Sex	0.381	1.253	0.304	0.762	−2.094…2.855
Age	0.461	0.630	0.731	0.466	−0.783…1.705
Angry	2.339	0.957	2.444	0.016	0.450…4.230
Sad	1.521	0.978	1.556	0.122	−0.410…3.452
Depression	0.154	0.140	1.095	0.275	−0.124…0.431
Sex × Depression	−0.181	0.181	−0.999	0.320	−0.539…0.177
Age × Depression	−0.002	0.100	−0.023	0.982	−0.200…0.196
Angry × Depression	−0.200	0.142	−1.404	0.162	−0.480…0.081
Sad × Depression	−0.049	0.144	−0.343	0.732	−0.333…0.234

#### Late Time Window 350–450 ms

The results of the mixed-effects regression model on mismatch responses in the late time window are presented in Table 4. In the late time window, the positive association between maternal depression symptoms and the mismatch response amplitudes of the child was nearly statistically significant [β = 0.289, 95% CI [–0.00819, 0.587], (SE 0.151), *p* = 0.0566]. If true, this means that the increase of one point in maternal EPDS sum scores predicted a + 0.29 μV change in the mismatch response amplitude of the child, resulting in decreased mismatch response amplitudes as maternal depression symptoms increased. In the angry emotion category, the interaction between the angry emotion and maternal depression symptoms was statistically significant [*Depression* × *Angry*: β = –0.315, 95% CI [–0.582, –0.0480], (SE 0.135), *p* = 0.0211, Bonferroni *p* = 0.0633], indicating that in the angry emotion category the increase of one point in maternal EPDS sum score predicted negligible –0.03 μV change in mismatch response amplitude of the child, taking into account the positive main effect of depression symptoms. The interaction between sex of the child and maternal depression symptoms was not statistically significant in this time window (*p* = 0.224).

**TABLE 4:** Results of the linear mixed-effects regression model (DF = 162) in the late time window (350–450 ms).

Parameter	β	SE	tStat	*p*-value	95% CI
Happy (Intercept)	−2.717	1.018	−2.668	0.008	−4.728…−0.706
Sex	−0.358	1.419	−0.252	0.801	−3.160…2.445
Age	0.672	0.715	0.940	0.349	−0.740…2.085
Angry	−1.435	0.920	−1.560	0.121	−3.253…0.382
Sad	−0.161	0.916	−0.175	0.861	−1.969…1.648
Depression	0.289	0.151	1.921	0.057	−0.008…0.587
Sex × Depression	−0.251	0.205	−1.221	0.224	−0.656…0.155
Age × Depression	0.065	0.114	0.572	0.568	−0.160…0.290
Angry × Depression	−0.315	0.135	−2.329	0.021	−0.582…−0.048
Sad × Depression	−0.214	0.135	−1.578	0.117	−0.481…0.054

However, when additional outlier values of the mismatch response amplitudes exceeding the value of 7 μV (*n* = 2) in the happy emotion category in the late time window were excluded from the analyses, the associations weakened below statistical significance.

**FIGURE 6:**
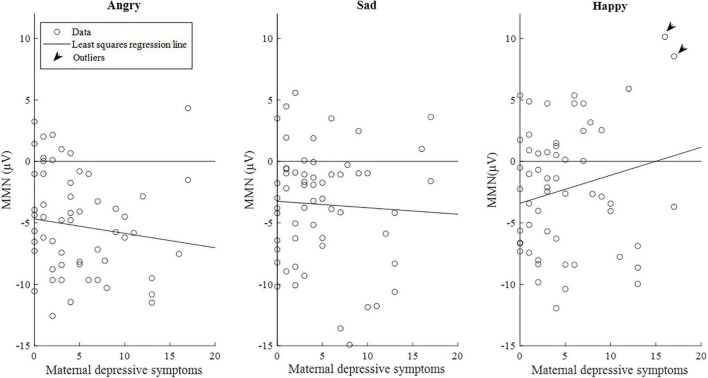
Scatterplots of maternal depression symptoms and the mismatch response amplitudes (μV) of the children (*n* = 58) in the late time window (350–450 ms). Of note, the lines are least-squares regression lines fitted across the data points, not the results of the mixed-effects models. Outliers = subjects with the mismatch response amplitude exceeding the value of 7 μV in the emotion category “Happy” (*N* = 2).

The correlations between maternal depression symptoms and the mismatch response amplitudes of the child in the late time window are presented in Figure 6. Supplementary Figure 2 shows the results separately for girls and boys.

## Discussion

The aim of this study was to investigate the association between the prenatal maternal depression symptoms and emotional sound perception of 3-year-old children as measured by mismatch responses. First, we hypothesized that maternal depression symptoms are associated with the amplitude of mismatch responses to emotional sounds in children. Second, based on previous studies (Otte et al., 2015; Lavonius et al., 2020), we hypothesized that this association depends on the valence of the emotion. In line with our hypothesis, we found a statistically significant association between prenatal maternal depression symptoms and mismatch response amplitude to emotional sounds. This association was found only in the early time window and only in happy emotional category – in sad emotional category (early time window) and in angry emotional category (late time window) the effect was reversed. No (1) significant main effects of maternal anxiety or its interactions with maternal depression symptoms, (2) main effects of sum score (maternal anxiety plus maternal depression symptoms), or (3) three-way effects (the interaction between maternal depression symptoms and emotion categories and sex or the interaction between the age of children, depression or emotion categories) were revealed in control analyses. In summary, our results provide neuropsychological evidence indicating that the children of mothers who have experienced prenatal depression symptoms may respond more weakly to happy sounds.

We are not aware of any previous studies using this study question in this age group with ERPs as a proxy of detection of different emotional stimuli. The studies investigating the relation of prenatal maternal stress and ERPs to emotional stimuli of the child have mainly been done in other age groups such as in infants (Otte et al., 2015; Lavonius et al., 2020). Also, studies using other EEG measures than ERPs, such as frontal asymmetry (Soe et al., 2016; Gustafsson et al., 2018; Goodman et al., 2021), exist, as well as studies using a visual stimulus (van den Heuvel et al., 2018). Correspondingly, previous studies have used some other indicators for prenatal stress rather than maternal depression symptoms to study the relation of maternal wellbeing during the pregnancy and emotional processing of the child. Using the same emotional sounds and the same birth cohort as in this study, Lavonius et al. (2020) studied the association between maternal sleep quality during pregnancy and the emotional processing of infants. They found, similar to the present results, an association between higher maternal sleep loss and decreased ERP amplitude for happy sounds in children. Furthermore, they found a parallel effect linking increased maternal sleepiness and smaller ERPs for happy sounds. These results provide further support for the hypothesis that deficiencies in maternal wellbeing during pregnancy are associated with children responding more weakly to happy stimuli and that these changes can be observed from infancy on into early childhood.

In this study, we used the mismatch response as an electrophysiological correlate of automatic auditory perception, not emotion processing or regulation. The fact that we found the association specifically in the early time window, typical latency for MMN, supports this interpretation. EEG does not allow us to spatially pinpoint the neural basis of the observed association, but during the emotion perception, a set of interacting brain regions (e.g., amygdala, insula, and orbitofrontal cortex) are activated (Lindquist et al., 2012). The neurodevelopmental structural changes in children related to exposure to prenatal maternal stress have been described in several brain areas, including temporal, frontal, and limbic regions (Adamson et al., 2018). Volume-specific (Wen et al., 2017) and functional connectivity (Soe et al., 2018) changes in the amygdala, the key structure involved in emotional processing, have been associated with prenatal maternal depression in two previous studies, especially in girls. Also, links between the structural features of amygdala and later emotional face processing in infancy have been described (Tuulari et al., 2020). One possible explanation for our findings could be that, in children of depressed mothers, happy sounds do not catch the attention as intensively as other sounds, or that children of depressed mothers have less experience and exposure to happy sounds. Overall, we can only speculate about the possible specific mechanism behind the association between maternal depression and decreased mismatch response amplitudes for happy stimuli in children discovered in our study sample, and further studies to investigate these mechanisms are required. Also, this effect may be modulated by child sex, which should be further assessed in future studies with larger sample sizes. Furthermore, it should be noted that our analyses are correlative and any causal relation between maternal depression and emotional mismatch responses of the child cannot be extrapolated. Effects of genetic factors or indirect (postnatal) environmental factors that correlate with maternal depression cannot be ruled out either.

### Strengths and Limitations

In this study, we examined EEG data from 56 three-year-olds. Our study would have benefited from a larger sample size as our study suffered from the large data attrition due to unexpected technical problems. However, studies are rare in this age group because behavioral measures are difficult to obtain (Putkinen et al., 2012).

Second, it should be noted that in this study the amount of trials was limited (a total 24 trials for each emotion variant per child). With a higher number of trials per condition, we might have been able to measure mismatch responses with higher precision, which in turn could have yielded a more reliable statistical inference. However, the multifeature MMN paradigm that we used allowed us to measure multiple deviants at once in a relatively short time period, increasing the odds to keep the child concentrated throughout the whole recording and, by that, decreasing the amount of possible movement artifacts. Third, we did not control the alertness level of the child before the EEG recording, which, therefore, might have influenced the differences in mismatch response amplitudes among children. It should also be noted that although low Apgar score and small gestational age were exclusion criteria in this study, data about possible disorders (e.g., autism spectrum disorders) or medication of the children were not collected.

Fourth, the emotional stimuli we used differed from standard and other deviants with respect to their emotional valence but also with respect to other sound properties, e.g., sound intensity and duration. It is possible that the mismatch responses we found in late time window (350–450 ms) are induced by the later syllable of/ta-ta/pseudoword. This point is difficult to assess further as Kostilainen et al. (2018) did not find similar late negative deflections for emotional sounds at their late time window (300–500 ms) in infants as we found now in 3-year-olds. Overall, it cannot be said for sure whether the changes we described above in mismatch response amplitudes of children result specifically from the emotional properties of the stimuli or from differences in the acoustic properties of the stimuli. Because such effect is very difficult to avoid, we assume that changes in mismatch response amplitude are related to emotional valence of the stimuli.

Fifth, our data set included many mothers who scored zero points in the EPDS questionnaire, and their children had almost a similar variance in mismatch responses compared to participants who exhibited associations to EPDS scores (Figures 5, 6). Similar spread of observations is featured in many studies (e.g., Mostert et al., 2016), and future studies should address the factors that explain the variance, e.g., biological samples or other prenatal exposures. Finally, our results did not survive Bonferroni correction, which means that the results should be considered preliminary and should be attempted to be replicated in future studies.

## Conclusion

We found a positive association between maternal prenatal depression symptoms and the emotional mismatch responses, indicating that brain responses of children exposed to elevated levels of maternal prenatal depression symptoms were weaker to happy sounds. This suggests that the 3-year-old children of mothers with depression symptoms may be less sensitive to automatically detect happy sounds compared to children whose mothers do not display symptoms of depression.

## Data Availability Statement

The datasets presented in this article are not readily available because for now, the data cannot be shared openly due to Finnish legislation and our ethical permission. Requests to access the datasets should be directed to SL, sisolu@utu.fi.

## Ethics Statement

The studies involving human participants were reviewed and approved by the Ethics Committee of the Hospital District of Southwest Finland. Written informed consent to participate in this study was provided by the participants’ legal guardian/next of kin. The child’s assent was also given.

## Author Contributions

SL and AR collected the EEG data. LK, HK, JT, ML, MH, and HA provided the study design. SL, HR, HA, and JT participated in the EEG data analyses and statistical analyses. SL, HR, and JT wrote the main manuscript. All authors reviewed and involved in critically revising the manuscript and approved the final version.

## Conflict of Interest

The authors declare that the research was conducted in the absence of any commercial or financial relationships that could be construed as a potential conflict of interest.

## Publisher’s Note

All claims expressed in this article are solely those of the authors and do not necessarily represent those of their affiliated organizations, or those of the publisher, the editors and the reviewers. Any product that may be evaluated in this article, or claim that may be made by its manufacturer, is not guaranteed or endorsed by the publisher.

## Footnotes

1
www.finnbrain.fi
