# Supplementary material for: Auditory Mismatch Responses to Emotional Stimuli in 3-Year-Olds in Relation to Prenatal Maternal Depression Symptoms
Source: Front Neurosci. 2022 May 17;16:868270. doi: 10.3389/fnins.2022.868270 (PMC9152314; doi:10.3389/fnins.2022.868270)
Supplement: Supplementary file 1 [file Data_Sheet_1.docx]

Supplementary Material

## Supplementary Tables

**Table S1.** Characteristics of mother-child dyads included in the analyses (n = 58). Apgar score = newborn health assessment scale 1-10 assessed 5min postnatally (Appearance, Pulse, Grimace, Activity, Respiration); BMI = body mass index; EPDS = Edinburgh Postnatal Depression Scale; gwk = gestational week; SCL-90 = Symptom Check-List. Some data is missing due to attrition in questionnaire and register data acquisition. ^a^ Half-siblings and different parent siblings also included.

|  |  |  | N | % | Mean | SD | Range |
| --- | --- | --- | --- | --- | --- | --- | --- |
| Children |  |  |  |  |  |  |  |
|  | Sex |  |  |  |  |  |  |
|  |  | Girl | 31 | 53.4% |  |  |  |
|  |  | Boy | 27 | 46.6% |  |  |  |
|  | Gestational weeks at birth |  |  |  | 39.8 | 1.44 | 36.3 – 42.3 |
|  | Birth weight (g) |  |  |  | 3598 | 544 | 2580 – 5470 |
|  | Apgar (5min) |  |  |  | 9.1 | 0.50 | 8 – 10 |
|  | Age at EEG recording (months) |  |  |  | 37.2 | 1.01 | 35.2 – 38.7 |
|  | Number of siblings (at age of 4y) ^a^ |  |  |  |  |  |  |
|  |  | 0 siblings | 10 | 17.2% |  |  |  |
|  |  | 1 sibling | 19 | 32.8% |  |  |  |
|  |  | 2 siblings | 2 | 3.4% |  |  |  |
|  |  | missing information | 27 | 46.6% |  |  |  |
|  | Day care status  (at age of 2y) |  |  |  |  |  |  |
|  |  | At home | 14 | 24.1% |  |  |  |
|  |  | Nursery school | 13 | 22.4% |  |  |  |
|  |  | Family day care | 6 | 10.3% |  |  |  |
|  |  | Shift nursery school | 1 | 1.7% |  |  |  |
|  |  | Missing data | 24 | 41.4% |  |  |  |
|  | Native language |  |  |  |  |  |  |
|  |  | Finnish | 56 | 96.6% |  |  |  |
|  |  | Swedish | 1 | 1.7% |  |  |  |
|  |  | Other | 1 | 1.7% |  |  |  |
| Mothers |  |  | 58 |  |  |  |  |
|  | Age at delivery (years) |  |  |  | 30.6 | 4.26 | 19 – 39 |
|  | Pre-pregnancy BMI  (kg/m^2^) |  |  |  | 24.5 | 4.34 | 19.0– 37.0 |
|  | Maternal depression symptoms (EPDS) |  |  |  |  |  |  |
|  |  | At gwk 24 |  |  | 4.93 | 4.63 | 0.0 – 17.0 |
|  |  | total score of 11-12 points in assessment | 2 | 3.4% |  |  |  |
|  |  | total score of 13-14 points in assessment | 3 | 5.2% |  |  |  |
|  |  | total score of > 14 points in assessment | 3 | 5.2% |  |  |  |
|  |  |  |  |  |  |  |  |
|  |  | 3 months postpartum |  |  | 4.34 | 4.07 | 0.0 – 16.0 |
|  |  | Missing data | 5 | 8.6% |  |  |  |
|  |  | 6 months postpartum |  |  | 4.63 | 5.12 | 0.0 – 23.0 |
|  |  | Missing data | 12 | 20.7% |  |  |  |
|  |  | 12 months postpartum |  |  | 4.46 | 4.57 | 0.0 – 19.0 |
|  |  | Missing data | 17 | 29.3% |  |  |  |
|  |  | 24 months postpartum |  |  | 4.40 | 4.07 | 0.0 – 14.0 |
|  |  | Missing data | 25 | 43.1% |  |  |  |
|  | Maternal anxiety  (SCL-90) |  |  |  |  |  |  |
|  |  | At gwk 24 |  |  | 3.82 | 4.34 | 0.0 – 16.7 |
|  |  | Missing data | 0 | 0.0% |  |  |  |
|  |  | 3 months postpartum |  |  | 2.66 | 3.35 | 0.0 – 15.0 |
|  |  | Missing data | 5 | 8.6% |  |  |  |
|  |  | 6 months postpartum |  |  | 2.76 | 4.91 | 0.0 – 28.0 |
|  |  | Missing data | 12 | 20.7% |  |  |  |
|  |  | 24 months postpartum |  |  | 2.88 | 3.90 | 0.0 – 16.0 |
|  |  | Missing data | 25 | 43.1% |  |  |  |
|  | Smoking during pregnancy |  |  |  |  |  |  |
|  |  | No smoking at all | 55 | 94.8% |  |  |  |
|  |  | Only before gwk 12 | 3 | 5.2% |  |  |  |
|  | Drinking during pregnancy |  |  |  |  |  |  |
|  | at gwk 12 | No | 47 | 81.0% |  |  |  |
|  |  | Every week | 2 | 3.4% |  |  |  |
|  |  | 1-2 times per month | 2 | 3.4% |  |  |  |
|  |  | Rarely | 3 | 5.2% |  |  |  |
|  |  | Missing data | 4 | 6.9% |  |  |  |
|  |  |  |  |  |  |  |  |
|  | at gwk 36 | No | 50 | 86.2% |  |  |  |
|  |  | Rarely | 4 | 6.9% |  |  |  |
|  |  | Missing data | 4 | 6.9% |  |  |  |
|  |  |  |  |  |  |  |  |
|  | Use of drugs during pregnancy | No | 54 | 93.1% |  |  |  |
|  |  | Yes | 0 | 0.0% |  |  |  |
|  |  | Missing data | 4 | 6.9% |  |  |  |
|  |  |  |  |  |  |  |  |
|  | Educational level | Matriculation examination or lower | 14 | 24.1% |  |  |  |
|  |  | Higher vocational training | 24 | 41.4% |  |  |  |
|  |  | University degree or higher | 20 | 34.5% |  |  |  |
|  |  |  |  |  |  |  |  |
|  | Monthly income (gwk 12) | < 500€ | 6 | 10.3% |  |  |  |
|  |  | 501-1000€ | 7 | 12.1% |  |  |  |
|  |  | 1001-1500€ | 8 | 13.8% |  |  |  |
|  |  | 1501-2000€ | 24 | 41.4% |  |  |  |
|  |  | 2001-2500€ | 10 | 17.2% |  |  |  |
|  |  | 2501-3000€ | 3 | 5.2% |  |  |  |
|  |  |  |  |  |  |  |  |

## Supplementary Figures

##
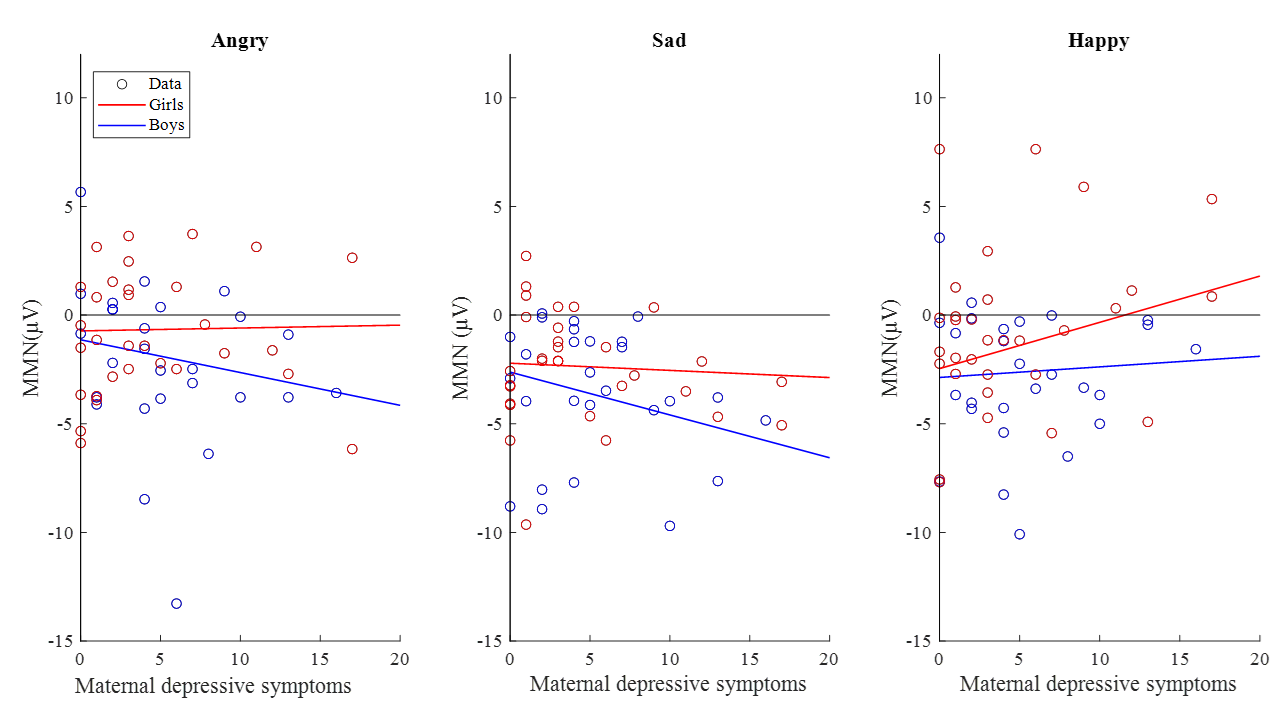
Figure S1. Results of the mixed-effects regression model in the early time window (80-120ms, n=58).

##
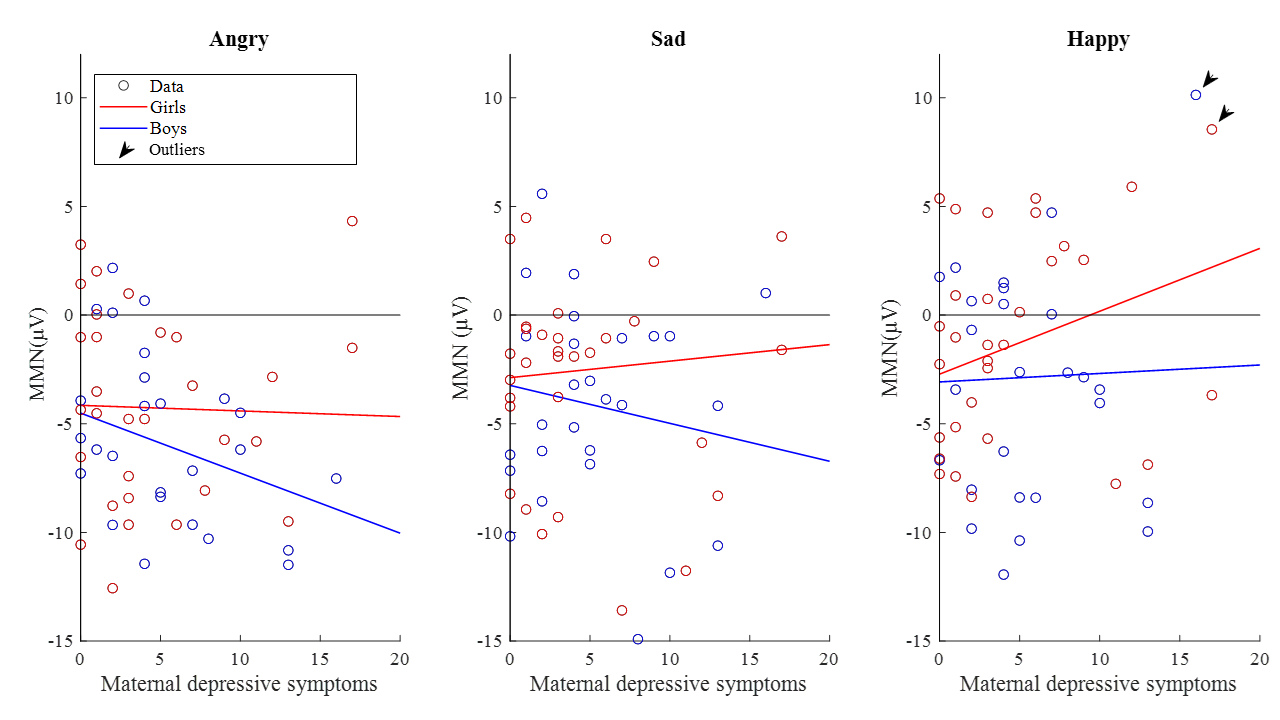


**Figure S2.** Results of the mixed-effects regression model in the late time window (350-450ms, n=58). Outliers = subjects with the MMN amplitude exceeding the value of 7µV in the emotion category “Happy” (N = 2).
